# Supplementary material for: Improved modeling of human vision by incorporating robustness to blur in convolutional neural networks
Source: Nat Commun. 2024 Mar 5;15:1989. doi: 10.1038/s41467-024-45679-0 (PMC10915141; doi:10.1038/s41467-024-45679-0)
Supplement: Supplementary file 1 — Supplementary Information [file 41467_2024_45679_MOESM1_ESM.pdf]

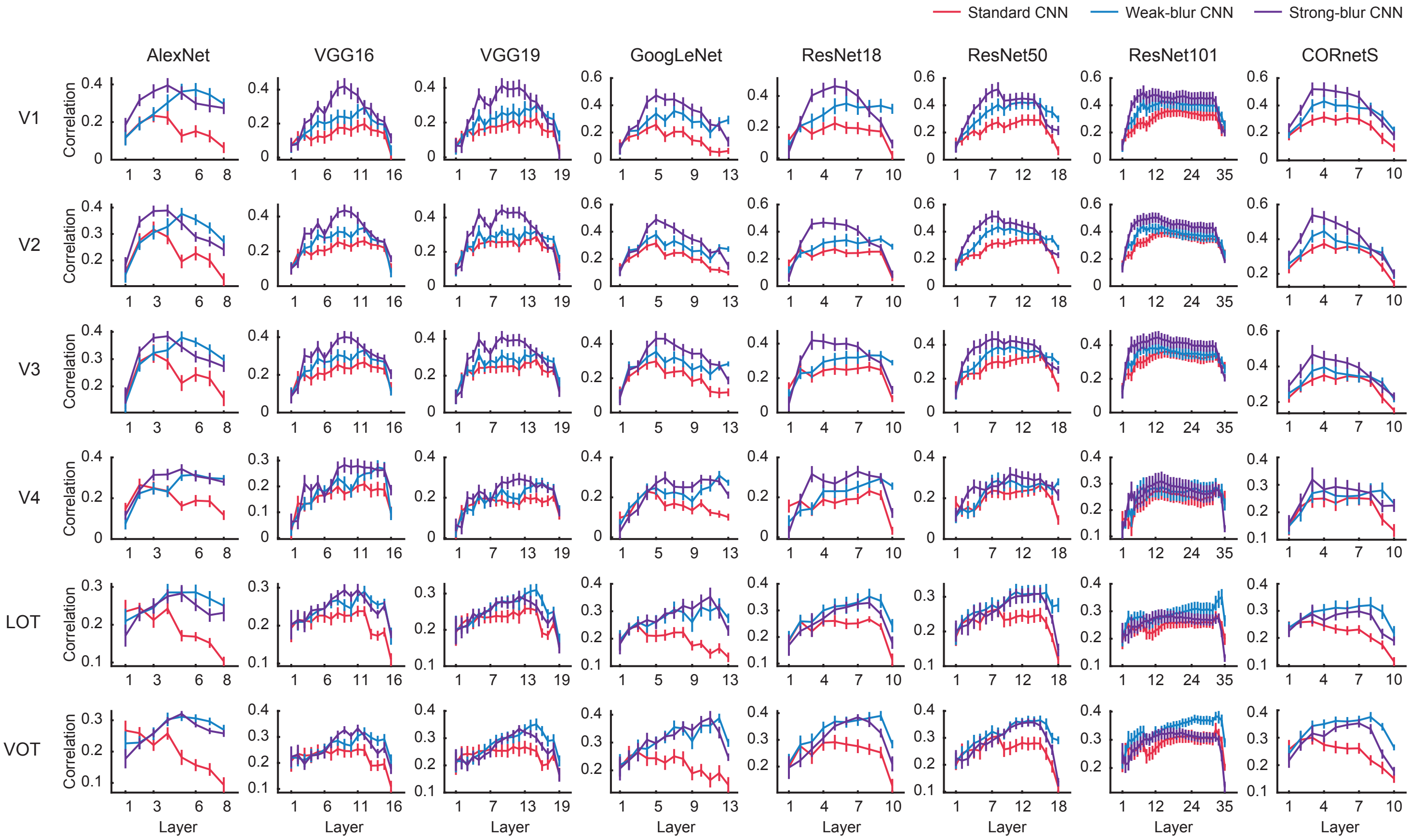

Supplementary Figure 1. Correlational similarity between CNN model responses and human neural responses to all viewing conditions, plotted for every CNN model by layer and for each visual area provided by Xu & Pashkam (2021).

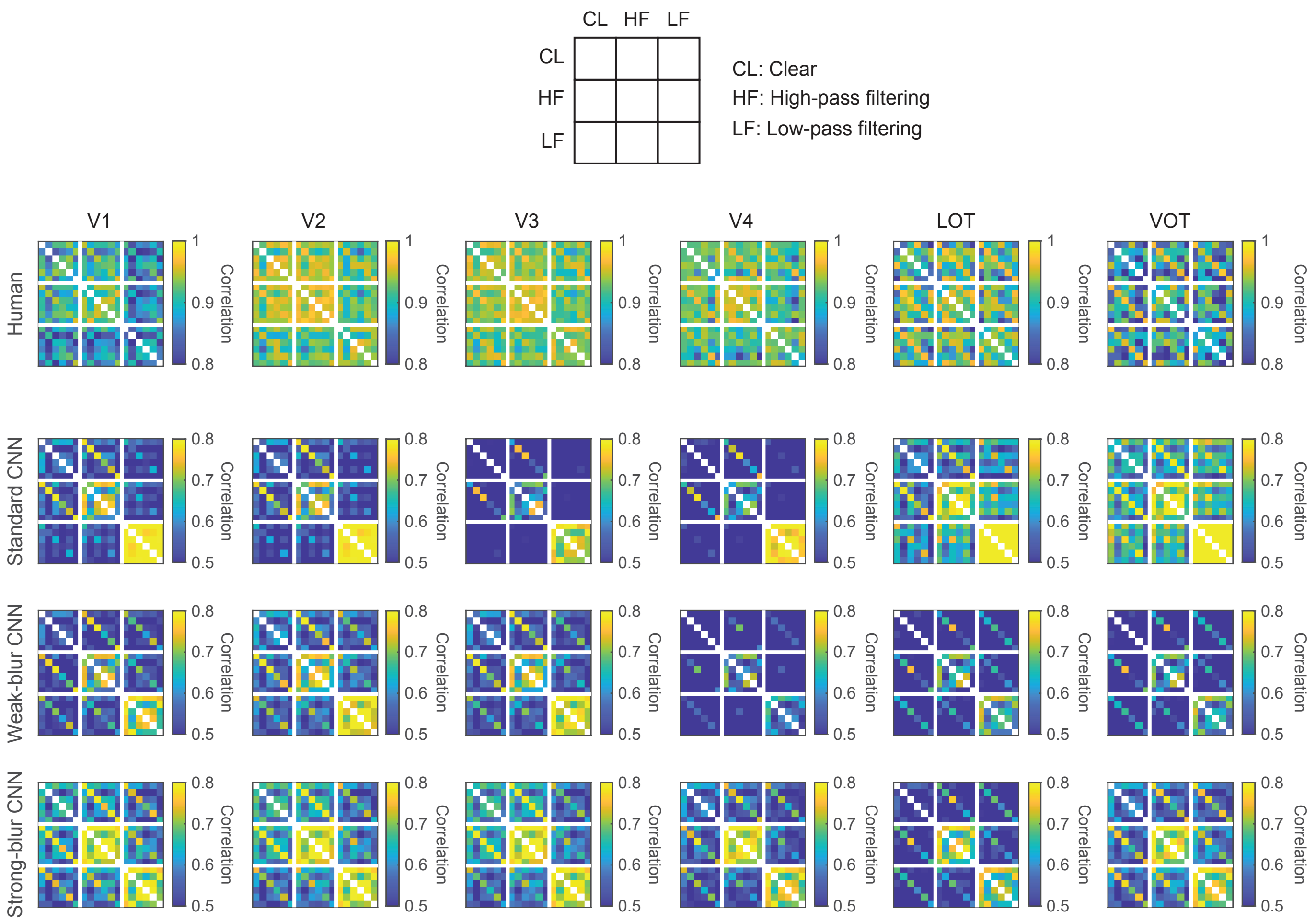

Supplementary Figure 2. Plots showing the mean Pearson correlational similarity matrices for human fMRI participants and CNNs in response to the clear, high-pass filtered and low-pass filtered object images used by Xu and Vaziri-Pashkam (2021). The CNN matrices show results for the layer that most accurately predicted neural activity in a specified human visual area.

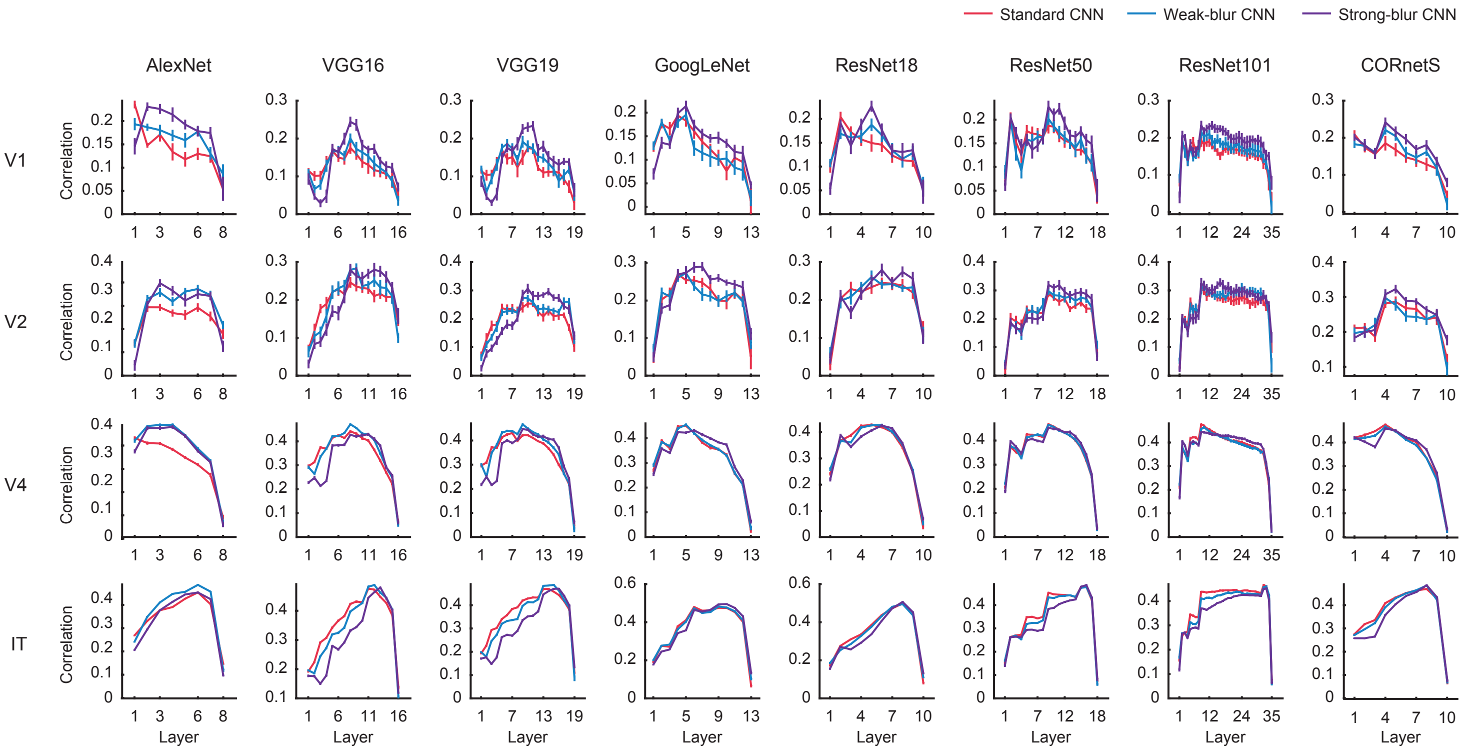

Supplementary Figure 3. Correlation between predicted and actual neuronal responses recorded from monkey V1, V2, V4 and IT, plotted for every CNN model by layer. Standard (red), weak-blur (blue) and strong-blur (purple) CNN analysis results are shown on each plot. Data from Schrimpf et al. (2020).

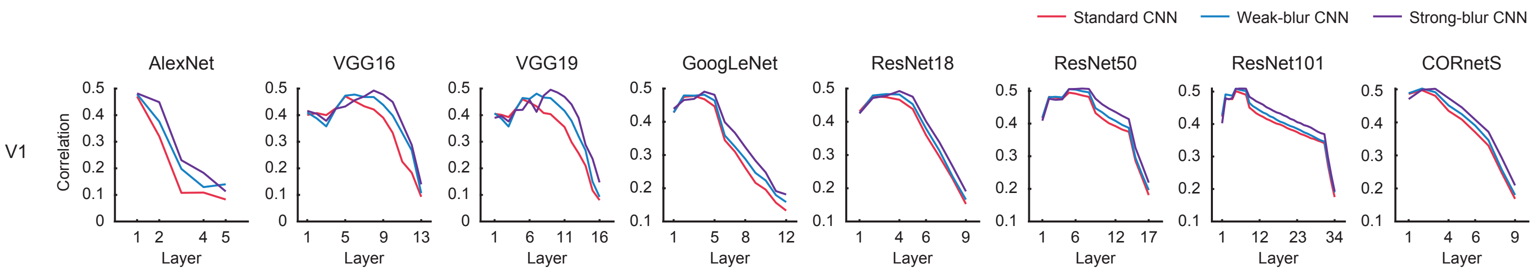

Supplementary Figure 4. Correlation between predicted and actual neuronal responses in macaque V1 to thousands of natural and synthetic images. Data obtained from Cadena et al. (2019).

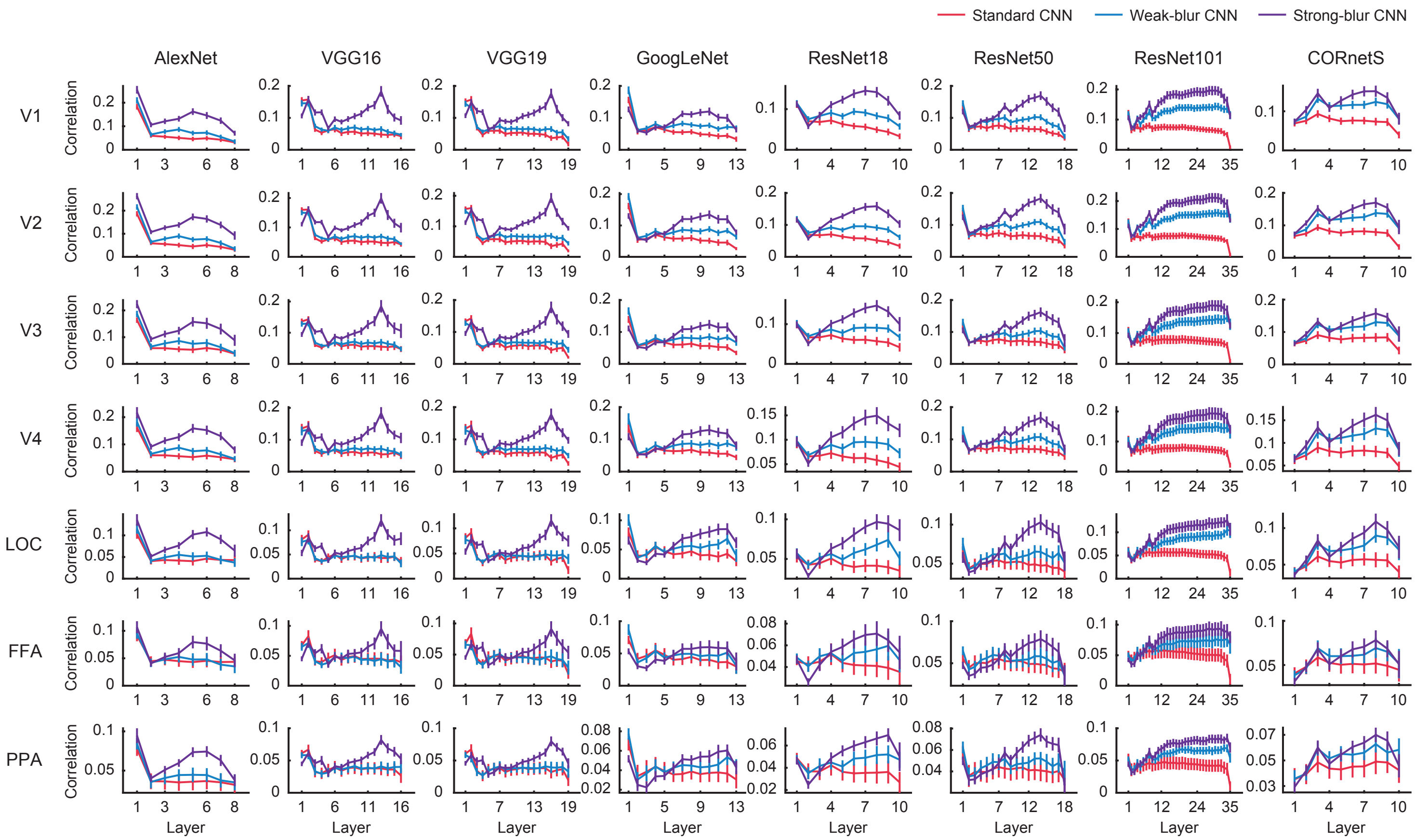

Supplementary Figure 5. Layerwise correlation of the RSA matrices between human observers and CNNs across brain regions in all viewing conditions combined (Jang et al., 2021). Standard CNNs (red), Weak-blur CNNs (blue), and Strong-blur CNNs (purple) were analyzed.

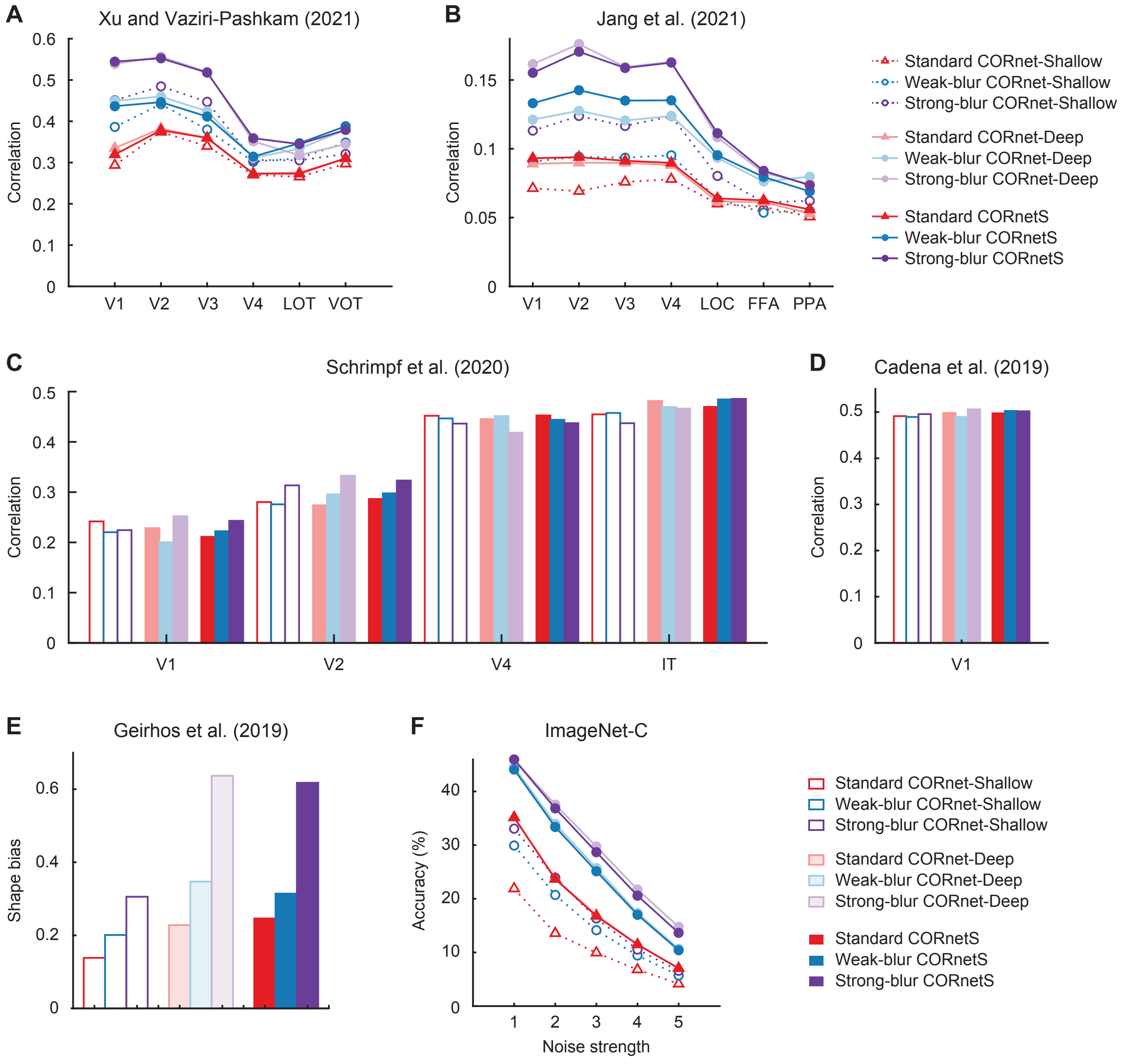

Supplementary Figure 6. Evaluation of blur training with a comparative analysis of recurrent neural network CORnet-S, control network CORnet-Shallow that lacks recurrent processing, and another feedforward control network, CORnet-Deep, which performs a matching number of non-linear operations as CORnet-S. Data obtained from **A** Xu and Vaziri-Pashkam (2021), **B** Jang et al. (2021), **C** Schrimpf et al. (2020), **D** Cadena et al. (2019), **E** Geirhos et al. (2019), and **F** ImageNet-C, Hendrycks and Dietterich (2019).

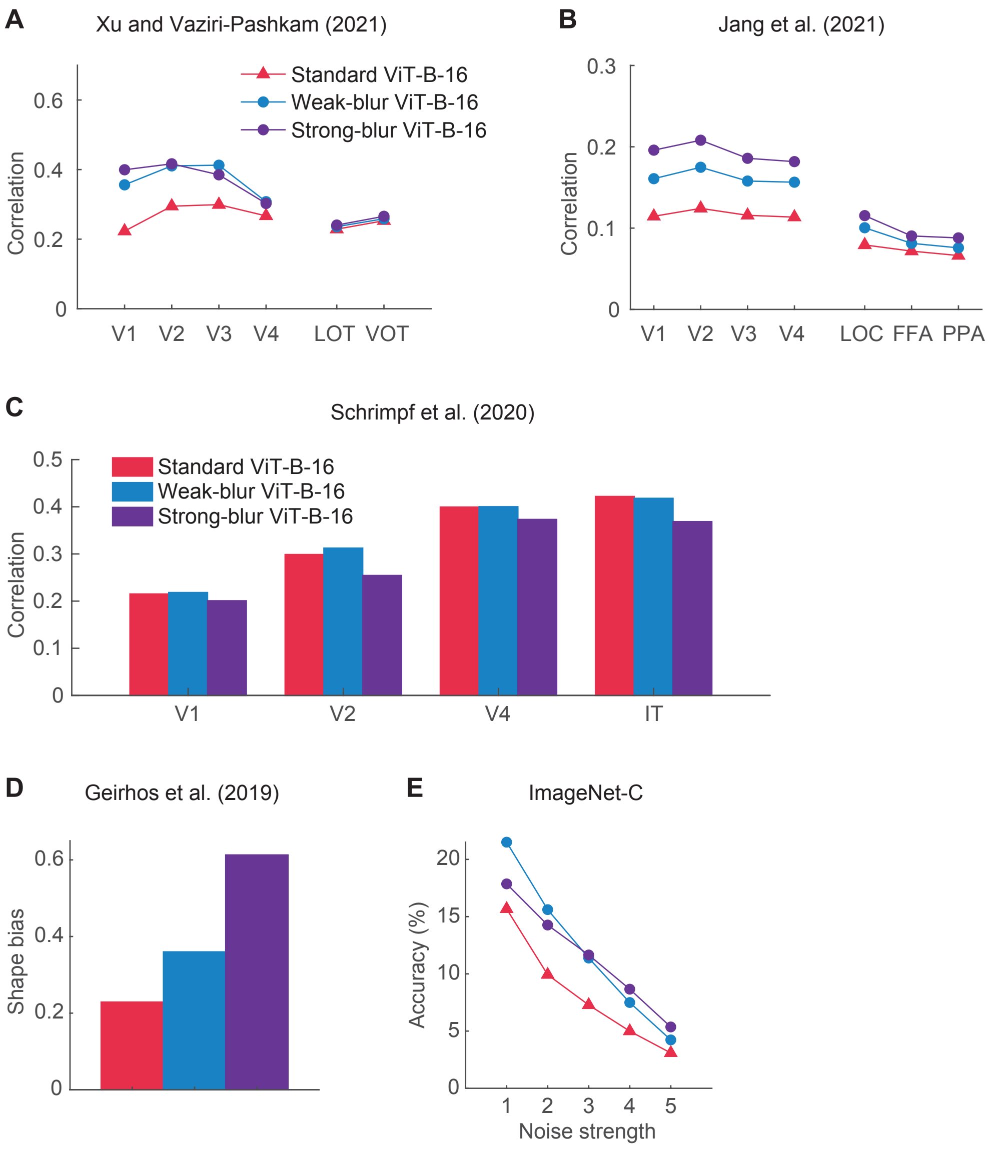

Supplementary Figure 7. Evaluation of blur training with visual transformer ViT-B-16. Data obtained from: **A** Xu and Vaziri-Pashkam (2021), **B** Jang et al. (2021), **C** Schrimpf et al. (2020), **D** Geirhos et al. (2019), and **E** ImageNet-C, Hendrycks and Dietterich (2019). The dataset from Cadena et al. (2019) was not included in the evaluation, as it required reducing the input image size to 40 × 40 pixels, which performs poorly with the ViT architecture.

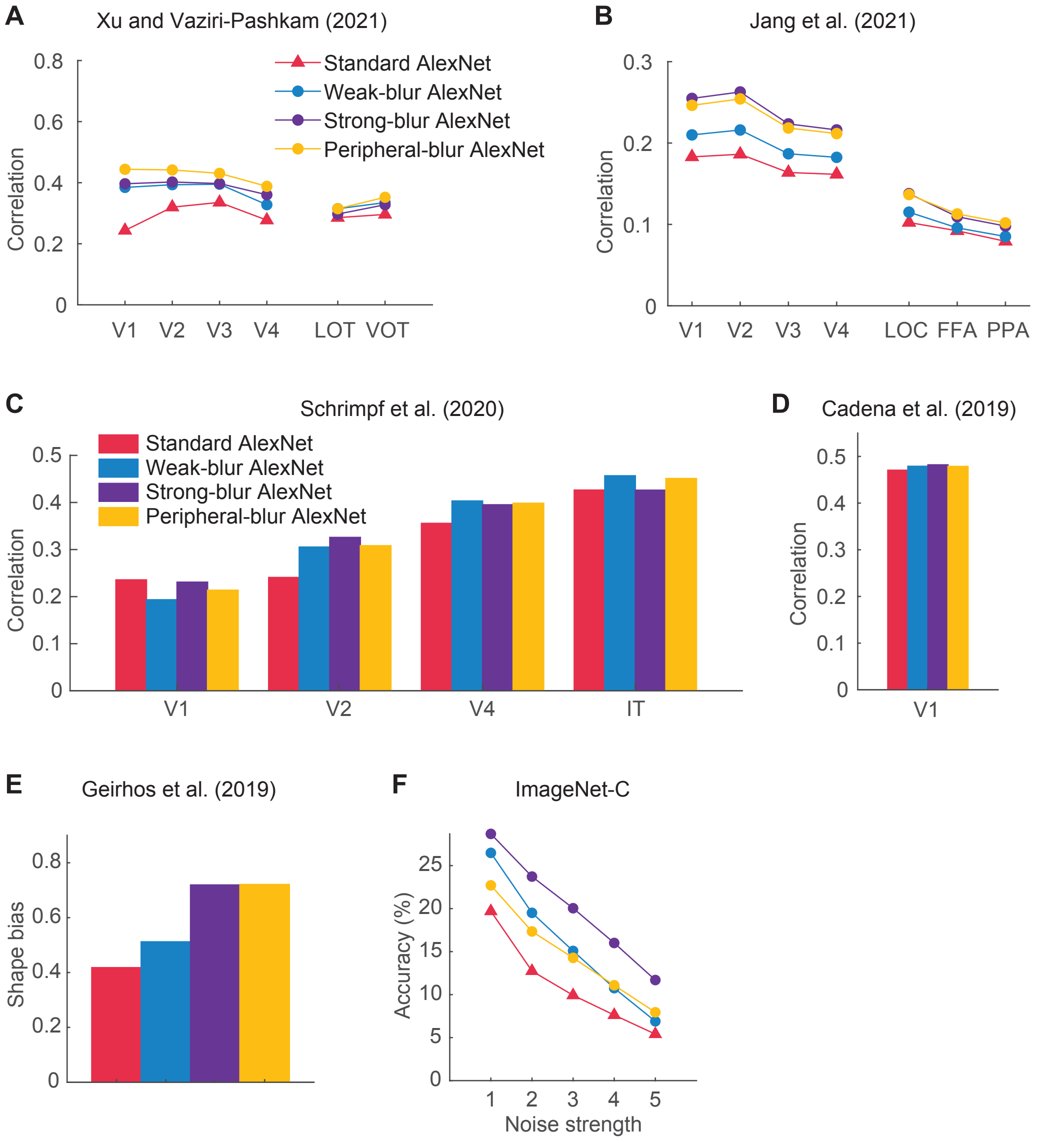

Supplementary Figure 8. Evaluation of peripheral blur training with AlexNet (yellow). Data obtained from: **A** Xu and Vaziri-Pashkam (2021), **B** Jang et al. (2021), **C** Schrimpf et al. (2020), **D** Cadena et al. (2019), **E** Geirhos et al. (2019), and **F** ImageNet-C, Hendrycks and Dietterich (2019).

CNN architecture	Number of sampled layers	Names of sampled layers
AlexNet	8	features_1, features_4, features_7, features_9, features_11, classifier_2, classifier_5, classifier_7
VGG16	16	Features_1, features_3, features_6, features_8, features_11, features_13, features_15, features_18, features_20, features_22, features_25, features_27, features_29, classifier_1, classifier_4, classifier_7
VGG19	19	features_1, features_3, features_6, features_8, features_11, features_13, features_15, features_17, features_20, features_22, features_24, features_26, features_29, features_31, features_33, features_35, classifier_1, classifier_4, classifier_7
GoogLeNet	13	conv1, conv2, conv3, inception3a, inception3b, inception4a, inception4b, inception4d, inception4e, inception5a, inception5b, fc_1
ResNet18	10	relu1, layer1_0, layer1_1, layer2_0, layer2_1, layer3_0, layer3_1, layer4_0, layer4_1, fc_1
ResNet50	18	relu1, layer1_0, layer1_1, layer1_2, layer2_0, layer2_1, layer2_2, layer2_3, layer3_0, layer3_1, layer3_2, layer3_3, layer3_4, layer3_5, layer4_0, layer4_1, layer4_2, fc_1
ResNet101	35	relu1, layer1_0, layer1_1, layer1_2, layer2_0, layer2_1, layer2_2, layer2_3, layer3_0, layer3_1, layer3_2, layer3_3, layer3_4, layer3_5, layer3_6, layer3_7, layer3_8, layer3_9, layer3_10, layer3_11, layer3_12, layer3_13, layer3_14, layer3_15, layer3_16, layer3_17, layer3_18, layer3_19, layer3_20, layer3_21, layer3_22, layer3_22, layer4_0, layer4_1, layer4_2, fc_1
CORnet-S	10	V1.output, V2.output.0, V2.output.1, V4.output.0, V4.output.1, V4.output.2, V4.output.3, IT.output.0, IT.output.1, decoder.output

Supplementary Table 1. Detailed layout of computational blocks within different networks for layerwise analysis.
